# Completeness of the Road-to-Health Booklet and Road-to-Health Card: Results of cross-sectional surveillance at a provincial tertiary hospital

**DOI:** 10.4102/sajhivmed.v19i1.765

**Published:** 2018-04-10

**Authors:** Harishia Naidoo, Theunis Avenant, Ameena Goga

**Affiliations:** 1 Department of Paediatrics and Child Health, Tembisa Provincial Tertiary Hospital, University of Pretoria, South Africa; 2 Department of Paediatrics, Kalafong Provincial Tertiary Hospital, University of Pretoria, South Africa; 3 Health Systems Research Unit, South African Medical Research Council, South Africa

## Abstract

**Background:**

Accurate record-keeping is important for continuity and quality of care. Completing a child’s Road-to-Health Booklet (RTHB), or the older, less detailed, Road-to-Health Card/Chart (RTHC), immediate interpretation thereof and appropriate action facilitates comprehensive care, which could contribute to a decline in child morbidity and mortality.

**Objective:**

This study aimed to assess the extent to which healthcare personnel working in catchment clinics of Kalafong Provincial Tertiary Hospital (KPTH), Tshwane district, South Africa, complete HIV-related, sociodemographic, neonatal, growth and immunisation information in the RTHC and/or RTHB.

**Methods:**

A cross-sectional, quantitative record review was conducted. Data were extracted from 318 RTHCs and/or RTHBs of children attending KPTH for paediatric care. Data extraction focused on six main areas, namely documentation of HIV-related, neonatal, sociodemographic, anthropometric, immunisation and vitamin A-related information. During data analysis, age-appropriate completeness scores were generated for each area and completeness of documentation in the RTHB and RTHC was assessed.

**Results:**

Data demonstrate significantly less unrecorded HIV-related information (maternal HIV status, timing of maternal HIV testing, timing of maternal antiretroviral therapy [ART] initiation, current maternal ART use and infant feeding decisions) in RTHBs compared with RTHCs (*p* < 001). Despite this, 24% of all RTHBs had no record of maternal HIV status and 67% of RTHBs from documented HIV-exposed infants had no record of maternal ART duration. Neonatal information completeness was similar between RTHBs and RTHCs, but socio-demographic completeness was significantly better in RTHBs compared with RTHCs (*p* = 0.006). Growth (especially weight), immunisation and vitamin A completeness was > 80% and similar between RTHBs and RTHCs. Length-for-age, weight-for-length and head circumference were plotted in < 5% of RTHBs and none of the RTHCs.

**Conclusion:**

Although completeness of key HIV-related information was better in RTHBs compared with RTHCs, RTHB completeness was suboptimal. Healthcare personnel need reminders to utilise the RTHB optimally to improve continuity and quality of child healthcare.

## Introduction

Accurate record-keeping is important for continuity and quality of care.^1^ Globally, patient-held maternal and/or child health records for literate and illiterate patients and healthcare personnel (including traditional birth attendants) have been used since the 1970s to track health status and document immunisation status.^2,3,4^ These records, though designed to track patient health histories and facilitate continuity of care amongst healthcare personnel, also empower patients to track their own health.

Internationally, the Road-to-Health Card/Chart (RTHC) is a useful patient-held child medical record as it summarises a child’s health in the first five years of life. The RTHC serves as a mobile databank.^3^ In some circumstances, particularly in populations with fragmented health services or migrating families, the RTHC may be the only reliable source of information. However, its usefulness depends on the knowledge, dedication and co-operation of mothers or caregivers and healthcare personnel.^5^

In South Africa, the leading causes of death amongst children aged less than five years include lower respiratory tract infection, diarrhoeal disease, malnutrition and perinatally acquired HIV.^6^ Better documentation of health status and risk factors, appropriate interpretation thereof and immediate action could reduce child morbidity and mortality. Healthcare personnel need to be vigilant for risk factors in order to provide comprehensive, relevant child healthcare.^7^

Road-to-Health Card/Charts have been used in South Africa since 1973. Over 40 different design formats were used until 1987 when a RTHC (Figure 1-A1)^8^ with a common design was implemented. The card mainly served as a record of immunisations and a growth monitoring chart. In 2010, the lack of continuity in HIV-related care prompted the design of a new Road-to-Health Booklet (RTHB) which dedicated two pages to HIV-related care. The RTHB (Figure 2-A1)^20^ also included more detailed growth monitoring such as head circumference measurements, length-for-age and mid-upper arm circumference, and included health promotion messages. Thus, compared with the RTHC, the RTHB serves as a more detailed patient-held child health record.

There have been few South African evaluations of this important health record, particularly since the inclusion of the HIV pages. Previous South African research on the RTHC was conducted in Kalafong Provincial Tertiary Hospital (KPTH) in 2005,^9^ Ga-Rankuwa township in 2007,^5^ the Vhembe District in 2010^7^ and the Makhado municipality, Limpopo.^10^ The KPTH study demonstrated that only 7% of doctors recorded information in the RTHC and only 54% of doctors asked mothers for the RTHC.^9^ The study by Tarwa et al. in Ga-Rankuwa demonstrated that health workers seldom asked to see the RTHC in primary and secondary settings. In addition, in 48% of consultations, the RTHC was not available.^5^ In Vhembe, only 14.3% of weights were accurately recorded.^7^ The study by Kitenge et al. in Makhado reported that nurses had poor interpretation of the growth curve; the immunisation section was the most utilised section. All four studies demonstrated that data recorded on the RTHC were mostly inaccurate, incomplete and not interpreted.^5,7,9,10^ Because of the absence of a designated space for specific HIV-related information on the RTHC, these studies did not include a detailed assessment of how HIV-related information is documented.

Previous South African research on use of the RTHB was conducted in the West Rand, Johannesburg, by Win in 2013, the Western Cape in 2014 and from an analysis of national survey data collected between 2011 and 2013. The study by Win in the West Rand reported that weight-for-age was completed better than the length-for-age and weight-for-length. Measuring of head circumference was missed in one-third of the children. A total of 41% of HIV-exposed children did not have information about HIV-polymerase chain reaction (PCR) testing.^11^ The Western Cape study, conducted in the Cape Town Metro, demonstrated good immunisation coverage, with 81% of caregivers presenting their RTHBs at consultations; however, only 56% of health workers asked for the RTHBs.^12^ The national surveys, conducted in 2011–2013, defined RTHB HIV-related completeness as documentation of infant birth weight, BCG immunisation, maternal syphilis status and maternal HIV status. Although it demonstrated an increase in RTHB completeness, from 23.1% (22% – 24%) in 2011–2012 to 43.3% (42.2% – 44.4%) in 2012–2013, completeness was still unsatisfactory by 2012–2013.^13^

Given this context, we aimed to gather detailed information on the extent to which healthcare personnel complete the prevention of mother-to-child transmission (PMTCT) and/or HIV, neonatal, sociodemographic, growth, immunisation and vitamin A sections in the RTHC and/or RTHB.

## Methods

We conducted a cross-sectional, quantitative review of data extracted from RTHCs and RTHBs of all children attending KPTH between May and December 2012, two years after RTHB implementation.

Kalafong Provincial Tertiary Hospital is situated in Tshwane district, 12 km west of the city centre of Pretoria, South Africa, next to the township of Atteridgeville. According to the 2011 census report, Tshwane district is the fifth most densely populated district in South Africa (2 921 488 people), and 23.2% of its population is under the age of 15 years.^10^ District antenatal HIV prevalence is approximately 25.2%.^11^ KPTH was one of South Africa’s largest regional hospitals before being designated as a provincial tertiary hospital; during 2012, it provided care for more than 13 000 paediatric outpatient visits and approximately 4500 paediatric inpatients (1243 short-stay admissions, 1182 non-neonatal inpatient admissions, 556 neonatal admissions, 612 kangaroo care admissions and 914 high care admissions; Department of Paediatrics, Annual report, 2012).

Children aged less than two years attending the paediatric outpatients department, paediatric short-stay ward and general paediatrics ward were eligible for enrolment in this study. We excluded children without a RTHC and/or RTHB, or who received care in the surgical wards or at the hospital casualty department, or whose parents did not provide consent.

Each enrolled child was allocated a unique, de-identified study identifier and no personal identifying information – name, address or hospital number – was documented. Data were extracted from the RTHC and/or RTHB onto individualised hard copy data collection sheets by one researcher (Dr Naidoo). The data collection sheet focused on how HIV-related information, sociodemographic details of the child and family, neonatal information, vitamin A supplementation, immunisations and growth parameters, were recorded on the RTHC and/or RTHB. Infant HIV-exposure was determined using the information documented on the RTHC and/or RTHB. For documented HIV-exposed infants, data on HIV-related care at birth, six and 10 weeks were extracted.

Parents and/or caregivers were not interviewed as the research focused on data extraction to assess completeness of the RTHC and/or RTHB.

Sample size was calculated to assess the completeness of HIV-related information in the RTHC or RTHB. From previous studies, it was expected that completeness would be around 35%.^13^ For this study, we wanted to estimate completeness at around 50% with a narrow range of ± 5% (50% ± 5%). With a confidence interval of 95%, the required sample size was 385 records (RTHCs and/or RTHBs).

All data were entered into Epi-Data version 3.1. Data were analysed using SAS version 9.4. Descriptive analyses were conducted for the entire study population, followed by comparisons between the RTHC and RTHB groups, using chi-square tests for categorical variables (Fisher’s exact test if expected cell count < 5) and *t*-tests or Wilcoxon rank sum tests for normally and non-normally distributed continuous variables, respectively.

During data analysis, continuous variables were generated by scoring responses that measured ‘HIV-related completeness’, ‘sociodemographic completeness’ and ‘neonatal completeness’ of the RTHC and/or RTHB.

HIV testing, sociodemographic and neonatal completeness were assessed for the entire study population, whilst PMTCT-completeness was only assessed for HIV-exposed children. HIV-related completeness was assessed using four composite variables: HIV testing completeness and PMTCT-completeness at birth, six weeks and 10 weeks. For PMTCT-completeness at birth, one point was allocated if each of the following was documented on the RTHC and/or RTHB: whether the mother was on antiretroviral treatment or not, receipt of feeding counselling, feeding decision, nevirapine prophylaxis given, maternal disclosure and partner testing. The maximum score measuring ‘fully complete’ was six. If the HIV-exposed infant was more than six weeks old, PMTCT-completeness at six weeks was assessed and one point was allocated if each of the following was documented on the RTHC and/or RTHB: feeding practice, feeding counselling, whether an infant HIV test was performed and if cotrimoxazole and nevirapine were prescribed. The maximum score (‘fully complete’) was five. If the HIV-exposed infant was more than 10 weeks old, PMTCT-completeness at 10 weeks was assessed and one point was allocated if each of the following was documented on the RTHC and/or RTHB: six-week HIV test result, post-test counselling conducted, cotrimoxazole and infant nevirapine prescribed. The maximum score was four. Thus, HIV-related completeness depended on whether the mother was HIV-positive and the age of the child.

Although the RTHC does not specifically list which HIV-related maternal and child details need to be captured, the point allocation was similar to that used for the RTHB as each point is pertinent for ongoing clinical management. For HIV completeness, attaining the maximum score at each time point was considered satisfactory, as HIV-related information is critical for ongoing care.

The secondary outcome of neonatal completeness was assessed by allocating one point to each of the following: birth weight, birth length, head circumference at birth, gestational age in weeks, mother’s blood group, mother’s RPR (rapid plasma reagin) result, antenatal history, intrapartum information, APGAR scores (an assessment of baby’s skin colour, heart rate, reflexes, muscle tone and respiration at at one, five and 10 minutes after birth) and neonatal feeding information. Thus, the maximum score was 10. A score of at least five or more was considered satisfactory for neonatal completeness.

Other outcomes of interest included sociodemographic completeness, which was assessed by allocating one point if each of the following was documented: date of birth, name of facility where child was born, who the child lives with, mother’s parity and the number of children currently alive. Thus, the maximum score was five. A score of more than three variables complete was considered satisfactory as this information is very important for ongoing care.

Our main hypothesis was that although HIV-related information would be more complete in the RTHB than in the RTHC, more than half of the RTHBs would have less than 50% of the required HIV-related information completed. We also hypothesised that: (1) there would be no difference in documentation of neonatal and sociodemographic information between RTHBs and RTHCs, (2) immunisations would be documented and given as per national protocol in > 90% children, with no difference between RTHBs and RTHCs, (3) weight and length-for-age would always be plotted but weight-for-length would be infrequently plotted in both RTHBs and RTHCs and (4) head circumference would not be documented for > 80% of children in RTHBs and RTHCs.

## Ethical consideration

Ethical approval was obtained from the Research Ethics Committee of the University of Pretoria. Written informed consent was obtained from parents before information from the RTHC and/or RTHB was extracted onto the data collection sheet.

## Results

Of the 318 children included in the study, 153 (48.1%) were inpatients and 165 (52.1%) were outpatients; 56 (17.6%) were HIV-exposed; 39.3% had RTHCs; and 60.7% had RTHBs (Figure 1).

**FIGURE 1:**
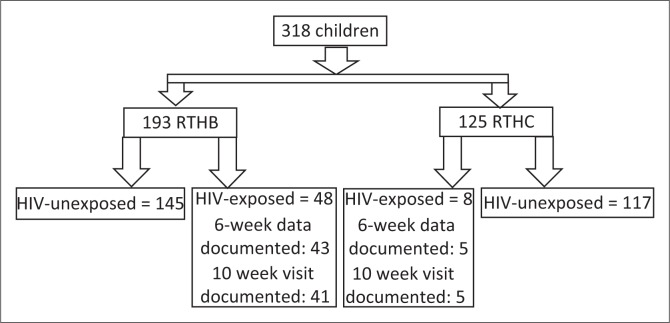
Study sample.

Children aged between 13 and 24 months comprised 10.9% of the RTHB population but 65.6% of the RTHC population (*p* < 0.001).

Data demonstrated significantly less unrecorded information on mother’s latest HIV test result, timing of maternal HIV testing, timing of ART initiation, current ART use and infant feeding decision in RTHBs compared with RTHCs. Despite this, 24% – 67% of RTHBs had unrecorded information for key HIV-related characteristics (Table 1). None of the RTHCs from infants born to HIV-positive mothers contained documented information on maternal ART use. Infant feeding decision by HIV-positive mothers was not documented in 37.5% of RTHBs and 62.5% of RTHCs.

**TABLE 1:** HIV-related information extracted from the Road-to-Health Card/Chart and/or Road-to-Health Booklet.

Background characteristics	RTHB		RTHC		*p*	Total	
	*N*	%	*N*	%		*N*	%
**Mother’s latest HIV result**							
Positive	48	24.9	8	6.4	< 0.001	56	17.6
Negative	98	50.8	28	22.4		126	39.6
Written in as a code	0	0	32	25.6		32	10.1
Missing	47	24.4	57	45.6		104	32.7
**When did the mother have the test?**							
Before pregnancy	15	7.8	0	0	< 0.001	15	4.7
During pregnancy	106	54.9	2	1.6		108	34.0
At delivery	5	2.6	5	4.0		10	3.1
Missing	67	34.7	118	94.4		185	58.2
**Amongst HIV-positive women, is the mother on lifelong ART?**							
Yes	20	41.7	0	0	< 0.001	20	35.7
No	16	33.3	0	0		16	28.6
Missing	12	25.0	8	100.0		20	35.7
**Amongst HIV-positive women, duration of ART at**							
Time of delivery	2	4.2	0	0	0.054	2	3.6
< 4 weeks	1	2.1	0	0		1	1.8
> 4 weeks but during pregnancy	13	27.1	0	0		13	23.2
Missing	32	66.7	8	100.0		40	71.4
**Decision about feeding amongst HIV-positive women**							
Exclusive breast	24	50.0	1	12.5	0.015	25	44.6
Exclusive formula	6	12.5	2	25		8	14.3
Missing	18	37.5	5	62.5		23	41.1

RTHB, Road-to-Health Booklet; RTHC, Road-to-Health Card/Chart; ART, antiretroviral therapy.

Amongst HIV-exposed infants, 45.3% of infant records (RTHC and/or RTHB) had maternal HIV test result and timing of maternal HIV test documented, with 65.8% within the RTHB group and 13.6% in the RTHC group (*p* < 0.0001; Table 2). Documentation of maternal ART use, maternal disclosure and partner testing was significantly better in RTHBs compared with RTHCs. Overall, completeness of HIV-related documentation was significantly better in RTHBs compared with RTHCs. A third (33%) of the RTHBs were fully complete with all six HIV-related characteristics (documentation of maternal ART use, receipt of infant feeding counselling, maternal infant feeding decision, maternal disclosure, infant nevirapine use and partner HIV testing, Table 2).

**TABLE 2:** Documentation and completeness of HIV-related information amongst HIV-exposed infants.

Background characteristics	RTHB		RTHC		*p*	Total	
	*N*	%	*N*	%		*N*	%
**HIV testing completeness**							
Nothing recorded	47	24.4	57	45.6	< 0.001	104	32.7
Test result or timing of test recorded	19	9.8	51	40.8		70	22.0
Test result and timing of test recorded	127	65.8	17	13.6		144	45.3
**Overall documentation of six key HIV-related characteristics**							
Total *N* (documented)	48	-	8	-	-	-	-
Mother on lifelong ART	36	75.00	0		< 0.001	36	64.29
Feeding counselling received	32	66.67	3	37.50	0.14	35	62.51
Decision about child feeding	30	62.50	3	37.50	0.252	33	58.93
Nevirapine given	29	60.42	3	37.50	0.252	32	57.14
Mother’s disclosure documented	26	54.17	0	-	0.005	26	46.43
Mother’s partner tested	24	50.00	0	-	0.008	24	42.86
**Overall completeness of HIV-related information for HIV-positive mothers**							
0 = nothing recorded	7	14.58	3	37.50	0.036	10	17.86
1 = one item recorded	6	12.50	2	25.00		8	14.29
2 = two items recorded	3	6.25	2	25.00		5	8.93
3 = three items recorded	3	6.25	1	12.50		4	7.14
4 = four items recorded	5	10.42	0	-		5	8.93
5 = five items recorded	8	16.67	0	-		8	14.29
6 = six items recorded	16	33.33	0	-		16	28.57
**Documentation at the 6-week visit for infants > 6 weeks old**							
Total *N* (documented)	43	-	5	-	-	-	-
Type of feeds documented	28	65.12	1	20	0.072	29	60.42
HIV-PCR documented	29	67.44	1	20	0.059	30	62.5
Cotrimoxazole initiation documented	19	67.44	1	20	0.059	20	41.67
Infant feeding was discussed	28	65.12	1	20	0.072	29	60.40
Nevirapine documented	27	65.79	0	-	0.012	27	56.25
**Completeness at the 6-week visit for infants > 6 weeks old**							
0 = nothing recorded	12	27.91	4	80	0.052	16	33.33
1 = one item recorded	2	4.65	0	-		2	4.17
4 = four items recorded	6	13.95	1	20		7	14.58
5 = five items recorded	23	53.49	0	-		23	47.92
**Documentation at the 10-week visit for infants > 10 weeks old**							
Total *N* (documented)	41	-	5	-	-	-	-
PCR result documented	19	46.34	0	-	0.067	19	41.30
Post-test counselling done	11	26.83	0	-	0.317	11	23.91
Cotrimoxazole given	10	24.39	0	-	0.570	10	21.74
Child received nevirapine	7	17.07	0	-	1.00	7	15.22
**Completeness of 10-week information for infants > 10 weeks old**							
0 = nothing recorded	22	53.66	5	100	0.684	27	58.70
1 = one item recorded	7	17.07	0	-		7	15.22
2 = two items recorded	2	4.88	0	-		2	4.35
3 = three items recorded	4	9.76	0	-		4	8.70
4 = four items recorded	6	14.63	0	-		6	13.04

RTHB, Road-to-Health Booklet; RTHC, Road-to-Health Card/Chart; ART, antiretroviral therapy; PCR, polymerase chain reaction.

Note: Documentation of birth weight, birth length, head circumference and APGARs were similar in the majority (90%) of RTHBs and RTHCs. However, documentation of mother’s blood group, antenatal and intrapartum history and neonatal feeding was better documented in the RTHB than in the RTHC (*p* < 0.000). Despite this, only 26% RTHBs had documented maternal antenatal history. There was no difference in the proportion of RTHBs and RTHCs that documented four or more neonatal characteristics, but maternal sociodemographic information was significantly better in RTHBs than in RTHCs, *p* = 0.0006 (Table 3).

**TABLE 3:** Completeness of the Road-to-Health Booklet and Road-to-Health Card/Chart for neonatal and sociodemographic information.

Characteristic	RTHB		RTHC		*p*	Total (318)	
	Number	%	Number	%		Number	%
**Neonatal information documented**							
Birth weight (g)	192	99.5	125	100	0.42	317	99.7
Birth length (cm)	174	90.2	115	92.0	0.35	289	90.9
Head circumference at birth (cm)	173	89.6	116	92.8	0.23	289	90.9
Gestational age in weeks	118	61.1	70	56.0	0.40	188	59.1
Mother’s blood group	123	63.7	39	31.2	0.001	162	50.9
Mother’s RPR	134	69.4	32	25.6	0.24	166	52.2
Antenatal history	51	26.4	3	2.4	< 0.001	54	17.0
Intrapartum	167	86.5	9	7.2	< 0.001	176	55.3
**APGAR documented**							
1 min	178	92.2	118	94.4	0.31	296	93.1
5 min	177	91.7	117	93.6	0.39	294	92.5
Neonatal feeding	182	94.3	0	0	-	-	-
**Neonatal feeding practice**							
EBF	146	75.6	2	1.6	< 0.001	148	46.5
EFF	12	6.2	0	0		12	3.8
MF	1	0.5	0	0		1	0.3
Other	1	0.5	0	0		1	0.3
Missing	33	17.1	123	98.4		156	49.1
**Completeness of neonatal and sociodemographic information**							
Neonatal information complete (> 4 characteristics completed)	179	92.7	116	92.8	0.30	299	94.0
Sociodemographic information complete (> 3 characteristics completed)	142	73.6	69	55.2	0.006	211	66.4

RTHB, Road-to-Health Booklet; RTHC, Road-to-Health Card/Chart; EBF, exclusively breastfed; EFF, exclusively formula fed; MF, mixed feeding.

At six weeks, completeness of documentation of HIV-related information was considerably better in RTHBs compared with RTHCs. A total of 54% of RTHBs were fully complete, with documentation of feeding decision and feeding discussion and PCR testing, cotrimoxazole initiation and nevirapine use. At 10 weeks, documentation and completeness of HIV-related information was poor in both RTHBs and RTHCs. None of the RTHCs had any documented information of 10-week HIV information and 54% of RTHBs had no documentation of 10-week HIV-related information (Table 2).

More than 80% RTHBs and RTHCs recorded immunisations, vitamin A and weight-for-age and plotted weight-for-age, with no significant differences between RTHBs and RTHCs (Table 4). However, only 3.6% RTHBs had head circumference documented and 5.2% had length-for-age recorded. No RTHC had head circumference, length-for-age and weight-for-length recorded or plotted.

**TABLE 4:** Immunisations, vitamin A and growth parameters.

Characteristic	RTHB		RTHC		*P*	Total
	*N*	%	*N*	%		
Immunisations up-to-date	155	80.3	101	80.8	0.71	256
Head circumference recorded at 14 weeks for children aged > 3 months	5	3.1	0	-	1.00	5
Vitamin A recorded in children aged > 6 months	90	92.8	84	97.7	0.18	174
Weight-for-age recorded at latest clinic visit	168	87.1	107	85.6	0.71	275
Length-for-age recorded	10	5.2	0	-	1.00	10
Weight-for-age plotted	163	84.5	106	84.8	0.55	269
Weight-for-length plotted	0	-	0	-	0	0

RTHB, Road-to-Health Booklet; RTHC, Road-to-Health Card/Chart.

## Discussion

This study demonstrates significantly better documentation of HIV-related information up to six weeks post-delivery, of maternal antenatal history, intrapartum history, neonatal feeding and sociodemographic information in RTHBs compared with RTHCs. Despite the significantly better recording of overall HIV-related information in RTHBs, more than half of the RTHBs from HIV-exposed infants had incomplete documentation of maternal ART use, feeding counselling, feeding decision, infant nevirapine use, maternal disclosure and partner HIV testing, and only 33% had all six HIV-related pieces of information recorded.

Documentation of maternal HIV-positive status in the RTHB correlated with the Tshwane district antenatal HIV prevalence of 25%.^14^ However, the RTHC grossly underestimated the district antenatal HIV prevalence at 6% owing to incomplete documentation. This probably relates to the lack of a specific section for documentation of the mother’s HIV status in the RTHC, and to the use of a code for maternal HIV status in RTHCs; thus, a percentage of positive mothers might have been hidden in the 26% ‘coded’ mothers’ results.

A study published in 2009, conducted in three clinics in KwaZulu-Natal, demonstrated that 86% of mothers supported the uncoded documentation of their HIV status in the patient-held RTHC and/or RTHB.^15^ They were of the opinion that documenting maternal HIV status would lead to better, faster and more appropriate care and treatment, with more openness and destigmatisation of HIV and/or AIDS. The remaining 14% of mothers were concerned about confidentiality as they had not disclosed their HIV status to anyone; thus, involuntary disclosure through the RTHC and/or RTHB was a concern. In our study, three-quarters (75.7%) of RTHBs and 28.8% RTHCs had documented maternal HIV status and only 54% RTHBs had documented maternal HIV disclosure. It is unclear whether the lack of documentation of maternal HIV status in the RTHB related to maternal fear or healthcare provider reluctance to test or document results. The reasons for poor documentation of disclosure are also unclear – did they relate to true non-disclosure or to fear of documentation of disclosure? The poor documentation of HIV-related information in RTHCs at six weeks and in RTHCs and/or RTHBs after 10 weeks is a cause for concern. A national situational assessment conducted in 2010 found that only 9% of immunisation service points provided routine infant HIV testing to infants with undocumented or unknown HIV status, 68% provided testing to HIV-exposed infants only and 15% integrated infant HIV testing into routine immunisation services.^16^ According to the South African National Department of Health Guidelines in 2010, all HIV-exposed infants should receive a PCR test at six weeks with the immunisation visit, and HIV-negative mothers should be retested during this visit. The identification of HIV-exposed uninfected infants is imperative to optimise interventions that prevent vertical transmission of HIV, such as maternal ART use, maternal viral suppression and breastfeeding under maternal ART cover. It has been shown that the HIV-infected infant has a better outcome if identified early.^17^ Reasons for inadequate infant HIV testing, identified during a national situational assessment, included differing policy guidelines owing to poor communication amongst managers, high workload, lack of training on the method of testing and lack of stock.^16^ The national RTHB analysis, using fewer variables to assess completeness, demonstrated an overall increase in completeness of the RTHB from 36% in 2010 to 43% in 2013; however, completeness in 2013 was still suboptimal. It was found that maternal and healthcare provider-related factors influenced completeness.^13^

The better documentation of neonatal feeding in the RTHB versus RTHC in this study could be explained by the presence of a dedicated space in the RTHB for feeding. Poor feeding practices, including inadequate breastfeeding, early introduction of poor-quality complementary food and failure to encourage children to eat, detrimentally influence child nutrition and health.^18^

We demonstrated significantly better documentation of key information such as antenatal and intrapartum history in the RTHB compared with the RTHC, supporting a previous study that the new format allowed for greater detail and better record-keeping in the RTHB.^14^ The 2005 KPTH study by Mulaudzi et al. reported that most of the child and family and neonatal information in the RTHCs was missing.^9^ Immunisations, record of vitamin A and plotting of weight were documented in more than 80% RTHC and/or RTHB. We hypothesised that immunisations would be given and documented in more than 90% of children. In our study, we found that immunisations were given and documented in 80% of children in both the RTHBs and the RTHCs. These figures are lower than Mulaudzi’s study which found 87% of immunisations to be up-to-date.^9^ There were only 4.6% missed immunisation opportunities in the Western Cape study, indicating good overall immunisation coverage. The main reasons for missed opportunities in the Western Cape study were incorrect knowledge regarding dosage schedules and contra-indications to certain immunisations.^12^ There is some evidence in Australia that the use of parent-held vaccination records is positively associated with being up-to-date with recommended vaccination schedule.^3^ In Australia, non-compliance with recommended vaccination schedules amongst children is associated with sociodemographic (e.g. low maternal education, low family socio-economic status and non-white ethnicity) and family-level factors (e.g. younger maternal age, large family size and late birth order). However, after controlling for sociodemographic and family factors, young American children (aged 19–35 months) with written parent-held vaccination records were significantly more likely to be up-to-date with scheduled vaccination.^3^

Vitamin A supplementation was documented in more than 90% of RTHBs and RTHCs. Mulaudzi’s study conducted in 2005 and/or 2006 demonstrated a 71% documentation of vitamin A. The 20% increase may be attributed to two reasons: (1) vitamin A supplementation and documentation thereof has become routine and (2) vitamin A campaigns were ongoing at local clinics during the time of this study.

Growth monitoring and promotion is considered the most useful tool in child health because regular growth monitoring enables the earlier detection of developmental and nutritional problems.^7^ In our study, weight-for-age was plotted in 85% of RTHC and/or RTHBs. This is similar to Win’s finding of weight-for-age plotted in 80% of RTHBs.^11^ However, length-for-age, contrary to our hypothesis, was plotted in only 5.2% of RTHBs, whereas weight-for-length was never plotted. Win’s study showed that length-for-age and weight-for-length was plotted in 37% and 33%, respectively. The slightly better plotting of measurements might be because Win’s study period was a year later with better training of personnel. Win’s study was also conducted in a different district area implying different skill levels amongst healthcare workers.^11^ A previous study showed that weight was plotted in 90% of RTHCs.^9^ Kitenge reported that 60% of nurses were unable to interpret the growth curve, indicating poor knowledge in identification of malnutrition. The nurses also did not understand the parameters of malnutrition and what a deviation in a growth curve implied. Challenges experienced in the Makhado study included staff shortages, work overload and lack of equipment.^10^

Chronic malnutrition (or stunting) is a major health problem amongst young children, accounting for more than 49% of all child deaths worldwide: globally, 33% of children under the age of five are stunted (low height-for-age), 27% are underweight (low weight-for-age) and in developing countries 9% are wasted (low weight-for-height).^19^ Thus, growth monitoring should be a critical component of child healthcare. The skill of accurately measuring and plotting weight must be matched by an ability to interpret it and act appropriately. Our data show that length is not being measured or plotted in a majority of the RTHBs. The measuring and plotting of length is a new parameter that was included in the RTHB, and it could be that this practice had not yet become routine. This necessitates further training and supervision of health workers to recognise and prevent stunting.^5^

Head circumference should be measured and plotted to detect normal growth and protein energy malnutrition that could affect the intellectual development of a child.^7^ In our study, only 3.6% of RTHBs documented the head circumference at 14 weeks despite it being specifically asked at the 14-week visit. Win’s study had more than one-third of head circumferences missed, which was significantly better than our study.^11^ We hypothesised that head circumference would not be documented for more than 80% of children, and our hypothesis proved to be true. Healthcare workers in previous studies were concerned about the added workload the new and more rigorous content in the RTHB would necessitate. However, a majority felt that there were more advantages than disadvantages.^15^ The lack of documentation of the head circumference might be related to these concerns.

Our study has several limitations. Firstly, it was conducted in one setting; thus, results may not be generalisable to the paediatric population in the rest of Gauteng province. However, the hospital is a large hospital serving 12 clinics, and results could represent quality of child health documentation within feeder clinics in the district. The RTHB or RTHC does not specify which clinic the child attended at each visit. This hampered our ability to rectify errors found in completion of these documents. The population of the HIV-exposed children was too small in the RTHC to conduct multivariable analysis. Lastly, by design, the study did not document availability of RTHC and/or RTHB during child health visits.

Based on our findings, we recommend in-service training and updates to healthcare workers on the use of the RTHB. In-service training will need to be goal directed to focus on areas of weakness. According to our study, this would include plotting and interpretation of length, weight-for-length, measuring of head circumference and focus on HIV-related information. Clearer guidelines and mentoring need to be available to healthcare workers for referral and support to children with malnutrition. The impact of a mentoring team to support, monitor and evaluate utilisation of the RTHB in each healthcare district should be investigated. This team can also provide health promotion messages and/or pamphlets in the common languages, from a higher, departmental level such as the Department of Health. A campaign can be launched to increase use and availability of the RTHB at every healthcare visit. This study confirms that use of the RTHB is a more user-friendly and comprehensive tool than the RTHC for completion of HIV-related information. A follow-on study would be useful to see which gaps persist after 2010 (our study) and 2011–2013 (national study). This would further assist in improving continuity and quality of care by assessing compliance with data completion in the RTHB. It might also be relevant to explore the relationship between completion of the RTHB and child well-being or morbidity.

## Conclusion

Our data demonstrate that although the RTHB with designated HIV sections is a more useful tool than the RTHC, it was completed suboptimally. This study provides evidence of incomplete documentation of health information in RTHBs and/or RTHCs, resulting in missed opportunities. The more comprehensive format of the RTHB offers a chance to improve documentation and reduce missed opportunities. There is a need to train and mentor healthcare personnel on optimal utilisation of the RTHB and to monitor RTHB completeness so that it may be used effectively as a curative, preventative and promotive tool in monitoring child health.

## Figures and Tables

**FIGURE 1-A1:**
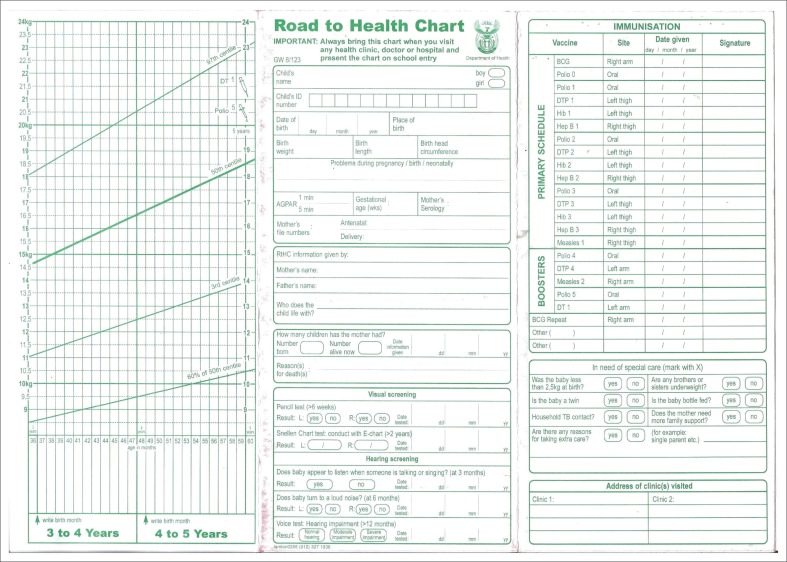
Road-to-Health Card.

**FIGURE 2-A1:**
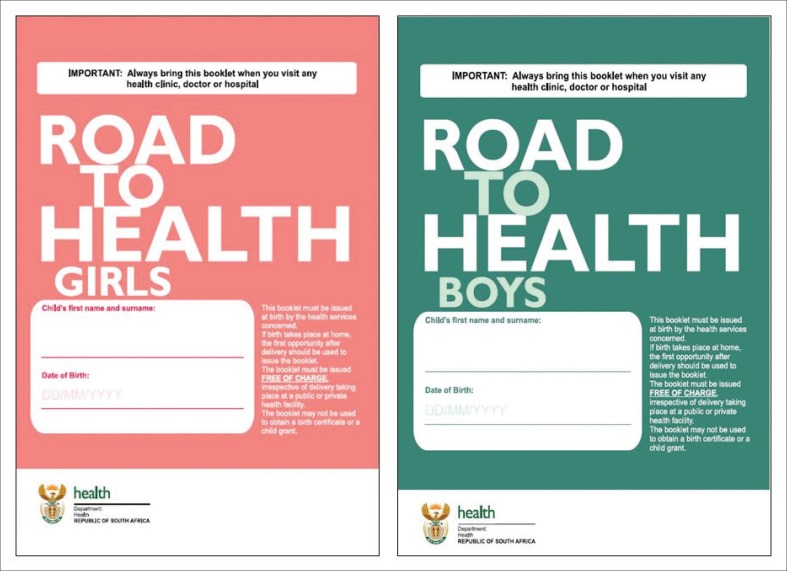
Road-to-Health Booklet.
